# A partial loss-of-function mutation in an Arabidopsis RNA polymerase III subunit leads to pleiotropic defects

**DOI:** 10.1093/jxb/erw020

**Published:** 2016-02-10

**Authors:** Kaeli C. M. Johnson, Yu Yu, Lei Gao, Ryan C. Eng, Geoffrey O. Wasteneys, Xuemei Chen, Xin Li

**Affiliations:** ^1^Department of Botany, University of British Columbia, Vancouver, V6T 1Z4, Canada; ^2^Michael Smith Laboratories, University of British Columbia, Vancouver, V6T 1Z4, Canada; ^3^Institute for Integrative Genome Biology, Department of Botany and Plant Sciences, University of California Riverside, Riverside, CA 92521, USA

**Keywords:** Alternative splicing, Arabidopsis, NRPC7, plant immunity, Pol III, RNA polymerase III, Rpc25.

## Abstract

Plants employ five DNA-dependent RNA polymerases (Pols) in transcription. One of these polymerases, Pol III, has previously been reported to transcribe 5S rRNA, tRNAs, and a number of small RNAs. However, in-depth functional analysis is complicated by the fact that knockout mutations in Pol subunits are typically lethal. Here, we report the characterization of the first known viable Pol III subunit mutant, *nrpc7-1*. This mutant was originally isolated from a forward genetic screen designed to identify enhancers of the autoimmune mutant *snc1*, which contains a gain-of-function mutation in a nucleotide-binding leucine-rich repeat (NLR) immune receptor-encoding gene. The *nrpc7-1* mutation occurs in an intron–exon splice site and results in intron retention in some *NRPC7* transcripts. There is a global disruption in RNA equilibrium in *nrpc7-1*, exemplified by the altered expression of a number of RNA molecules, some of which are not reported to be transcribed by Pol III. There are developmental defects associated with the mutation, as homozygous mutant plants are dwarf, have stunted roots and siliques, and possess serrated leaves. These defects are possibly due to altered small RNA stability or activity. Additionally, the *nrpc7-1* mutation confers an *NLR*-specific alternative splicing defect that correlates with enhanced disease resistance, highlighting the importance of alternative splicing in regulating NLR activity. Altogether, these results reveal novel roles for Pol III in maintaining RNA homeostasis, adjusting the expression of a diverse suite of genes, and indirectly modulating gene splicing. Future analyses using the *nrpc7-1* mutant will be instrumental in examining other unknown Pol III functions.

## Highlight

Analysis of the first known viable RNA polymerase III subunit mutant in Arabidopsis reveals novel roles for this complex in maintaining RNA homeostasis, adjusting the expression of a diverse suite of genes, and indirectly modulating alternative splicing.

## Introduction

Transcription under both static and dynamic conditions requires the action of evolutionarily conserved multi-subunit enzymes known as DNA-dependent RNA polymerases (Pols). All eukaryotes possess three distinct RNA polymerases (Pols I, II, and III), each of which transcribes specific suites of genes (Cramer *et al.*, 2008).

Pol I transcribes 45S rRNA, which is the precursor to 5.8S, 18S and 25S rRNAs. Pol II transcribes mRNAs as well as most small nuclear (sn)RNAs and micro (mi)RNAs. Pol III was previously thought to be primarily required for the transcription of ‘housekeeping’ genes such as those encoding 5S rRNA and tRNAs. However, recent reports indicate that the Pol III transcriptome is more diverse than formerly assumed (Dieci *et al.*, 2007). There are two additional plant-specific RNA polymerases, Pol IV and Pol V, which are required for the biogenesis and functional activity of small interfering (si)RNAs (Haag and Pikaard, 2011). As knockout mutations in the genes encoding the subunits of Pols I, II, and III are lethal, there is a dearth of functional analysis of plant Pols.

While there are a number of published studies examining global transcriptomic changes in plants under various conditions (e.g. Nagano *et al.*, 2012, Woo *et al.*, 2012, Zhu *et al.*, 2013), the literature to date has largely focused on the roles played by Pol II-transcribed RNAs in regulating plants’ responses to stimuli. Stimulus-induced alteration of expression of protein-coding genes has been extensively documented. Numerous recent reports have highlighted the importance of miRNAs in regulating a broad spectrum of biological processes including development (Wu, 2013), flowering time (Spanudakis and Jackson, 2014), drought stress (Ding *et al.*, 2013), metal toxicity (Gupta *et al.*, 2014), immunity (Staiger *et al.*, 2013), and phytohormone crosstalk (Curaba *et al.*, 2014), among others. Furthermore, the biosynthesis, functional mechanisms, and degradation pathways of miRNAs have been well studied (Rogers and Chen, 2013).

Comparatively little is known about Pol III-transcribed RNAs and how they aid plants in responding to intrinsic and extrinsic signals. An RNA molecule with significant sequence and structural similarity to 5S rRNA was found to regulate alternative splicing of certain pre-mRNAs in Arabidopsis (Hammond *et al.*, 2009). Intriguingly, studies in a variety of eukaryotes indicate that Pol III-transcribed non-coding RNAs may play regulatory roles in addition to their housekeeping functions (Hu *et al.*, 2012).

Among the various stimuli to which plants are subjected, biotic stress in the form of pathogenic infection requires that plants be able to respond rapidly and initiate signalling cascades specific to the type of pathogen being encountered. While plants possess physical barriers and broad spectrum resistance that is activated by conserved features of pathogenic microbes, many pathogens are able to deliver infection-promoting effector molecules into the plant cell, thereby bypassing this layer of plant immunity (Bigeard *et al.*, 2015). However, the plant genome contains a large number of genes encoding nucleotide-binding leucine-rich repeat proteins (NLRs; also referred to as Nod-like receptors due to their structural similarity to mammalian proteins of the same name), which either directly bind to pathogenic effectors or detect their activities within the plant cell in a highly specific manner (Li *et al.*, 2015). Upon recognition of its cognate effector, NLR activation results in rapidly induced and robust defence responses. Plant NLRs can be sorted into two classes based on their N-termini: some possess a Toll-interleukin 1 receptor (TIR) domain and are thus termed TNLs, while others contain a coiled-coil (CC) domain and are referred to as CNLs.

NLR-mediated signalling must be tightly controlled under both resting and induced conditions, as improper signalling through this pathway may lead to either enhanced disease susceptibility or autoimmunity. However, the regulatory mechanisms underlying NLR-mediated signalling are only partially understood. A successful forward genetic suppressor screen previously conducted in our lab used the gain-of-function autoimmune TNL mutant *suppressor of npr1, constitutive 1* (*snc1*; Li *et al.*, 2001; Zhang *et al.*, 2003) to search for positive regulators of immunity (Johnson *et al.*, 2012). More recently, we have undertaken a forward genetic screen to identify negative regulators of NLR-mediated immunity.

Here, we report the characterization of *nrpc7-1*, a partial loss-of-function allele of the gene encoding the Arabidopsis orthologue of yeast Rpc25, a Pol III subunit. This mutant was isolated from our MUSE (MUTANT, *snc1*-ENHANCING) forward genetic screen conducted in the *modifier of snc1 4* (*mos4*) *snc1* double mutant background. A null mutation in *NRPC7* is lethal, while a mutation in an intron–exon splice site junction gives rise to intronic retention in some *NRPC7* transcripts, resulting in viable mutant plants. While the *nrpc7-1 mos4 snc1* triple mutant displays enhanced resistance against the virulent oomycete pathogen *Hyaloperonospora arabidopsidis* (*H.a.*) Noco2, the *nrpc7-1* single mutant exhibits wild type-level resistance. This correlates with the altered splicing of *SNC1* observed in the triple mutant but not in the single mutant. Morphologically, the *nrpc7-1* mutant is dwarf and has serrated leaves, short roots, and stunted siliques, although flowering time does not appear to be affected. The expression and potentially activity of a number of RNAs are distorted in *nrpc7-1*, contributing to its developmental defects. In keeping with its known function, we observed that the NRPC7 protein localizes to the nucleus. This is the first reported viable Pol III subunit mutant in Arabidopsis.

## Materials and methods

### Plant growth conditions and mutant isolation

Plants were grown either on soil or on half-strength Murashige and Skoog (MS) medium supplemented with 1% sucrose and 0.3% phytagel. All plants were grown under long day conditions (16h light–8h dark) at 22 ºC in climate-controlled chambers. The *muse4* mutant was isolated from the MUSE screen, described previously (Huang *et al.*, 2013).

### Total RNA extraction and analysis

Approximately 0.1g tissue was collected from 2-week-old seedlings grown on ½ MS, and the Ambion ToTALLY RNA Total RNA Isolation Kit (Thermo Fisher Scientific) was used to extract total RNA. For the comparison of rRNA levels, total RNA was run on a 2% agarose gel. To reverse transcribe 0.4 μg RNA to cDNA, the Reverse Transcriptase M-MLV (Takara) was used after treating the RNA with DNaseI (Promega). The sequences of primers used were: 4F 5′-AATCTCCCTCTCGAAGATGC-3′ and 4R 5′-AAAGGCTTTGCGTCCTCTGC-3′ for *MUSE4*/*NRPC7*; U1F 5′-TACCTGGACGGGGTCAAC-3′ and U1R 5′-CCCTCTGCCA CAAATAATGAC-3′ for *U1*; U2F 5′-TCGGCCCACACGAT ATTAAC-3′ and U2R 5′-GCAGTAGTGCAACGCATAGG-3′ for *U2*; 5SF 5′-GGATGCGATCATACCAGC-3′ and 5SR 5′-GAGGGA TGCAACACGAGG-3′ for *5S* rRNA; 7SLF 5′-CAAATCAAGTG GTTCAACCC-3′ and 7SLR 5′-CTTCGACGTTATCATCTGCG-3′ for *7SL* RNA; GlnF 5′-GGTTCTATGGTGTAGTGGTTAGC-3′ and GlnR 5′-TACCGGGAGTCGAACCCAG-3′ for *tRNA-Gln*; GlyF 5′-GCACCAGTGGTCTAGTGGTA-3′ and GlyR 5′-TGCACCAGCCGGGAATCGAA-3′ for *tRNA-Gly*; and LeuF 5′-TGTCAGAAGTGGGGTTTGAACC-3′ and LeuR 5′-TCAGGATGGCCGAGTGGTCTAA-3′ for *tRNA-Leu*. Primers used for amplification of *SNC1*, *RPS4*, *SR30*, *PAD4*, and *ACTIN7* were previously described (Zhang *et al.*, 2003; Cheng *et al.*, 2009; Xu *et al.*, 2012).

### Infection assays

*H*.*a*. Noco2 infection was performed by spraying 2-week-old soil-grown seedlings with a spore suspension with a concentration of 10^5^ spores per millilitre of water. Inoculated seedlings were grown for 7 d at 18 ºC in a growth chamber with ~80% humidity and a 12h light–12h dark cycle. Sporulation was then quantified using a haemocytometer to count the number of spores from five plants shaken in 1ml of water. Five replicates were performed for each of three independent trials. *P*.*s*.*m*. ES4326 infection was performed by infiltrating the abaxial leaf surface of 4-week-old soil-grown seedlings with bacteria suspended in 10mM MgCl_2_ (OD_600_=0.0005). Leaf punches were collected at day 0 and day 3, and serial dilutions were performed and plated on LB medium. Plates were incubated at 28 ºC for 24h before colony forming units were measured.

### Positional cloning and Illumina whole-genome sequencing

Positional cloning of *muse4* was performed by crossing the *muse4 mos4 snc1* triple mutant (generated in the Col-0 ecotype) with wild type Landsberg *erecta*. Twenty-four F2 plants homozygous for all three mutations were used for crude mapping, and approximately 500 F3 plants homozygous for *mos4* and *snc1* and heterozygous for *muse4* were used for fine mapping. The markers used in mapping were derived from insertion/deletion polymorphisms between the Col-0 and L*er* Arabidopsis ecotypes (Jander *et al.*, 2002; http://www.arabidopsis.org). After determining that the mutation must be located on the top of chromosome 1 between 1.4 MB and 2.75 MB, extracted genomic DNA from *muse4 mos4 snc1* was sequenced using the Illumina sequencing platform.

### Preparation of transgenic plants and confocal microscopy

Full length *At1g06790* genomic DNA, including 766bp upstream of the start codon, was amplified via PCR, cloned into the pCambia1305 vector, and transformed into *muse4 mos4 snc1* using the floral dip method (Clough and Bent, 1998). The full length genomic fragment was also cloned into a pCambia1305 vector containing a green fluorescent protein (GFP) tag. Transgenic plants were selected for on ½ MS plates containing 50mg ml^–1^ hygromycin. Confocal images of wild type (negative control), 35S::X-GFP (positive control), and *NRPC7-GFP* transgenic seedlings were obtained using a PerkinElmer Ultraview VoX spinning disc confocal system (PerkinElmer) mounted on a Leica DM16 000 B inverted microscope and equipped with a Hamamatsu 9100-02 electron multiplier CCD camera (Hamamatsu). An argon 488nm laser line with a complementary (522/36) emission band-pass filter to detect GFP or a 561nm laser with a complementary (595/50) emission band-pass filter to detect propidium iodide was used. Images were acquired with a ×63 (water) objective lens. To stain the nuclei and the cell wall, seedlings were incubated in a 10 µg ml^–1^ solution of propidium iodide (Calbiochem) for 1min, rinsed with water, and mounted on a slide and coverslip prior to imaging.

### Yeast complementation

Full length *MUSE4* cDNA was cloned into the yeast expression vector p425-GPD with primers 5′-CGCggatccATGTTT TATCTTAGCGAGC-3′and 5′- ACGCgtcgacTCACTCTTCTTG ATCAACC-3′, using *Bam*HI and *Sal*I digestion sites. *MUSE4* and empty vector control plasmids were introduced into the yeast *rpc25-ts* strain using a standard polyethylene glycol–lithium acetate yeast transformation protocol (http://labs.fhcrc.org/gottschling/Yeast%20Protocols/ytrans.html). Yeast transformants were grown overnight, serially diluted, and plated onto SD-Leu plates grown under either 28 or 37 ºC to assay for growth.

### Small RNA library construction and sequencing

Small RNAs within the size range of 15–40 nt were fractionated from total RNAs by 15% polyacrylamide gel electrophoresis. These small RNAs were then ligated with the 3′ and 5′ adapters sequentially using the Small RNA Sample Preparation Kit (Illumina) according to the manufacturer’s instructions. A reverse transcription reaction followed by a low cycle PCR was performed to obtain final products for deep sequencing. The wild type and *muse4* libraries were barcoded and sequenced in one channel on an Illumina Hiseq2000.

### Analysis of small RNA high throughput sequencing Data

PERL scripts were used to process small RNA raw reads as per Lertpanyasampatha *et al.* (2012). To summarize, reads were passed through Illumina’s quality control filter before being sorted into bins based on their barcodes and having their adaptor sequences removed. SOAP2 was used to map reads within the size range of 20–24 nt to the Tair10 Arabidopsis genome (Li *et al.*, 2009). Differential small RNA regions were identified as previously described (Dinh *et al.*, 2014). For analysis of differentially expressed miRNAs, all known Arabidopsis miRNAs were downloaded from miRBase (Release 20 from www.mirbase.org; Griffiths-Jones *et al.*, 2008). PERL scripts were used to determine the expression level of known miRNAs in the small RNA libraries and then normalize these counts to reads per million (RPMs). miRNAs with <10 RPMs in both *nrpc7-1* and wild type libraries were removed. The differentially expressed miRNAs were identified by comparing expression in the *nrpc7-1* library with wild type. The Audic–Claverie method was used to calculate *P*-values (Audic and Claverie, 1997), which were subsequently adjusted as described by Benjamini and Hochberg (1995) to determine the false discovery rate (FDR). To qualify as a differentially expressed miRNA, both a fold change ≥2 between wild type and *nrpc7-1* and an FDR <0.05 were necessary.

## Results

### The isolation, characterization, and identification of the *muse4*/*nrpc7-1* mutant

The MUSE screen was designed to identify enhancers of the dwarf autoimmune mutant *snc1* and has been described previously (Huang *et al.*, 2013). To avoid potential lethality resulting from dramatically enhanced autoimmunity, the *snc1* suppressor *mos4* was included in the genetic background of the screen. Seeds from the wild type-like *mos4 snc1* plants were mutagenized with ethyl methanesulfonate, and the M2 population was screened for plants displaying a reversion to *snc1*-like morphology and resistance. A number of mutant lines were isolated, one of which (*muse4*) was selected for further characterization. When the triple mutant was backcrossed to *mos4 snc1*, all progeny appeared wild type-like, indicating that *muse4* is a recessive mutation.

**Fig. 1.**
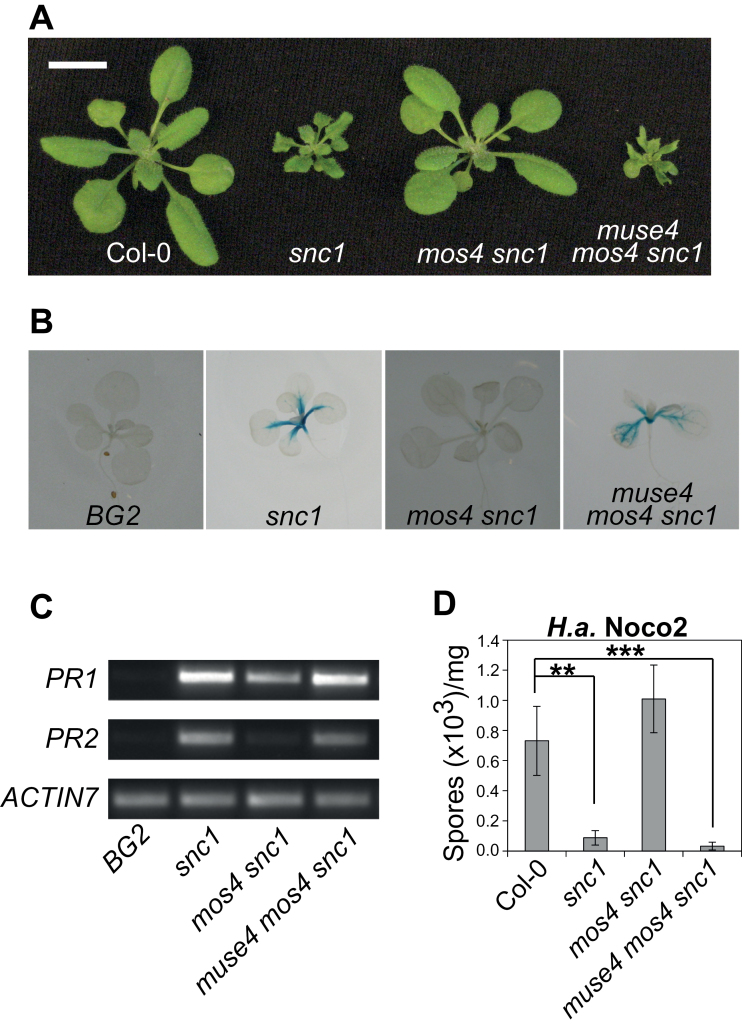
Characterization of the *muse4 mos4 snc1* triple mutant. (A) Morphology of soil-grown plants of the indicated genotypes, photographed 4 weeks post-germination. Scale bar represents 1cm. (B) *pPR2-GUS* expression in seedlings of the indicated genotypes grown on 1/2 MS medium for 10 d. (C) *PR1* and *PR2* gene expression in the noted genotypes, as determined by qPCR. *ACTIN7* expression serves as a loading control. (D) Growth of *H*.*a*. Noco2 on indicated genotypes 7 d post-inoculation with 1×10^5^ spores innoculum ml^–1^. Values represent the average of 4 replicates of 5 plants each ±SD. **P≤0.01; ***P≤0.001.

As shown in Fig. 1A, the *muse4 mos4 snc1* plants exhibit dwarf, curled leaf morphology similar to that observed for *snc1* plants. In addition, the *muse4 mos4 snc1* plants have serrated and slightly chlorotic leaves. The *muse4* mutation also re-establishes the constitutive expression of the defence marker *PATHOGENESIS-RELATED* (*PR*) genes observed in *snc1* but absent in *mos4 snc1*. A *pPR2-GUS* reporter gene construct was used to visualize *PR2* gene expression in seedlings, and GUS staining was much stronger in the triple mutant than in *mos4 snc1* (Fig. 1B). Consistent with this observation, qPCR demonstrated that expression of *PR1* and *PR2* is elevated in the triple mutant (Fig. 1C).

To examine whether the *muse4* mutation alters resistance to the virulent oomycete strain *Hyaloperonospora arabidopsidis* Noco2, 2-week-old triple mutant seedlings were spray-inoculated with this pathogen. The enhanced resistance observed in *snc1* but lost in *mos4 snc1* was found to be reconstituted in the triple mutant (Fig. 1D). Together, these data indicate that *muse4* restores all examined *snc1*-like phenotypes in the *mos4 snc1* background.

**Fig. 2.**
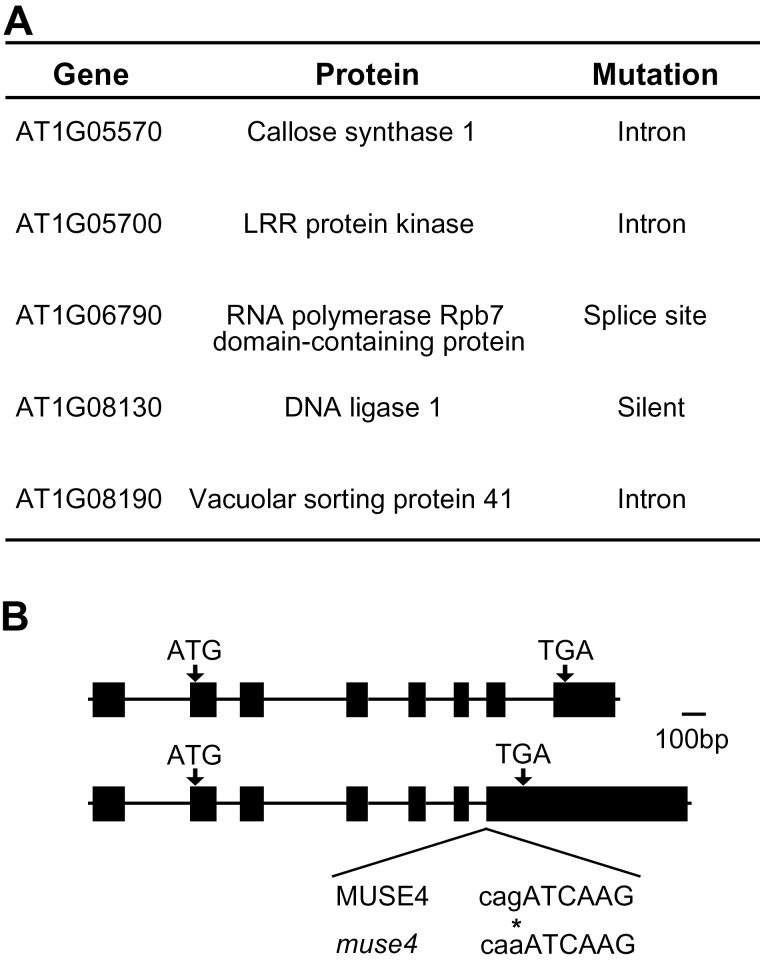
Map-based cloning of the *muse4* locus on chromosome 1. (A) Point mutations identified within the mapping region from Illumina sequencing of the *muse4-1 snc1 mos4* triple mutant. Two independent mutations in introns were identified in AT1G05570. (B) The two alternatively spliced variants of *MUSE4* and the position of the molecular lesion in *muse4-1* (*nrpc7-1*) and *muse4-2* (*nrpc7-2*). Boxes and lines represent exons and introns, respectively.

A positional cloning strategy was employed to determine the molecular lesion responsible for the observed phenotypes. The *muse4 mos4 snc1* mutant, which was generated in the Col-0 ecotype, was crossed to Landsberg *erecta* (L*er*). From the F2 population, 24 plants displaying the triple mutant morphology were selected for crude mapping, which identified a linkage to the top of chromosome 1. Several F2 plants heterozygous at the top of chromosome 1 (but homozygous for *snc1* and *mos4* to prevent interference by these loci) were used to generate a fine mapping population of approximately 500 plants. The mutation was narrowed down to between the markers T7A14 (1.4 MB) and F22O13 (2.75 MB). Genomic DNA was extracted from *muse4 mos4 snc1* triple mutant plants and sequenced using the Illumina whole-genome sequencing platform. The sequencing results were compared with the Arabidopsis reference genome, and five genes in this region were found to contain mutations (Fig. 2A). The mutations in three of these genes are located in introns and the mutation in one gene was found to be silent, therefore the mutation in the remaining gene (*At1g06790*) was selected as the most likely candidate for *muse4*. This gene encodes the Arabidopsis orthologue of the yeast Pol III subunit Rpc25, NUCLEAR RNA POLYMERASE C, SUBUNIT 7 (NRPC7; Ream *et al.*, 2015), and the *muse4* mutation is at the intron–exon junction just before the sixth exon (Fig. 2B).

### The mutation at an intron–exon junction of *NRPC7* results in intron retention and is responsible for the *muse4* phenotypes

**Fig. 3.**
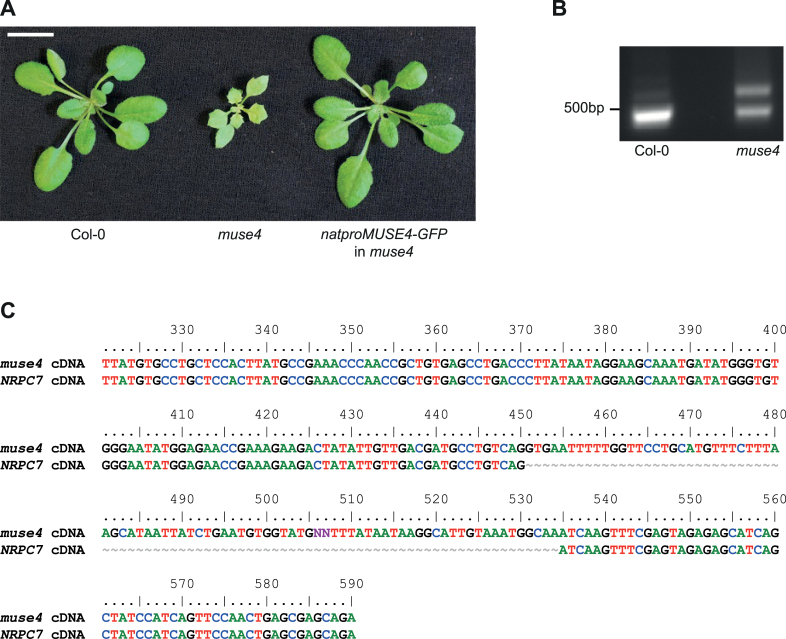
*MUSE4* is *NRPC7*. (A) MUSE4 tagged with GFP and expressed under the control of its native promoter is able to complement the *muse4* single mutant defects. Plants were grown on soil for 3 weeks. (B) The size of the *MUSE4* transcript in wild type and *muse4* was examined using cDNA reverse-transcribed from total RNA. (C) The larger *muse4* band in (B) was excised, purified, and sequenced, and found to retain the intron preceding the intron–exon splice site mutation in *muse4*.

To verify that the mutation in *NRPC7* is responsible for the *muse4* phenotypes, a full-length wild type copy of the gene driven by its native promoter and fused to the *GFP*-encoding gene at its 3′ end was transformed into the single mutant, which was generated by backcrossing the triple mutant to Col-0 and selecting plants homozygous for wild type *SNC1* and *MOS4* that retained the serrated leaf phenotype and dwarf size. Eight independent T2 lines displayed wild type morphology, and one representative line can be seen in Fig. 3A. These data suggest that *NRPC7* can fully complement the *muse4* phenotypes, and therefore that *MUSE4* is indeed *NRPC7*.

We hypothesized that the *muse4* mutation in the intron–exon junction of *NRPC7* results in retention of the preceding intron. To test this, we designed primers flanking the intron of interest and amplified cDNA from wild type and *muse4*. A strong band of the expected size (465bp) was observed in wild type while in *muse4* two bands were observed, one of the expected size and one slightly larger (Fig. 3B). The larger band was excised from the gel and the PCR product was purified and sequenced. As predicted, sequencing revealed that the larger band corresponded to a transcript in which the intron preceding the *muse4* mutation had been retained (Fig. 3C).

Despite strong sequence similarity between NRPC7 and known Rpc25 proteins in other species (Supplementary Fig. S1 at *JXB* online), the *NRPC7* gene failed to complement a temperature-sensitive *rpc25* yeast knockout line (Supplementary Fig. S2), suggesting divergence between the plant and yeast NRPC7.

It is expected that a knockout mutation in *NRPC7* would be embryo lethal, as a previous study showed that loss-of-function mutations in RNA polymerase subunits are not transmitted maternally (Onodera *et al.* 2008). Indeed, when we let the heterozygous *nrpc7* T-DNA insertion line CS1001213 self-fertilize and then planted the progeny, we identified 23 wild type plants lacking the insertion, 46 heterozygotes, and 0 plants that were homozygous for the insertion, matching the expected 1:2:0 (wild type:heterozygote:homozygote) ratio for a lethal mutation. We also performed reciprocal crosses between this heterozygous T-DNA insertion line and *muse4* and found that none of the F1 progeny contained the T-DNA insertion, indicating that the T-DNA/*muse4* heterozygotes are not viable. These results, combined with the data in Fig. 3B showing that *muse4* still produces some properly spliced transcripts without intron retention, as well as the fact that *muse4* is a recessive mutation, suggest that *muse4* is a partial loss-of-function allele of *NRPC7*. Therefore, we renamed *muse4* as *nrpc7-1* and the T-DNA allele as *nrpc7-2*.

### Splicing of *SNC1* is altered in the *nrpc7-1 mos4 snc1* background

**Fig. 4.**
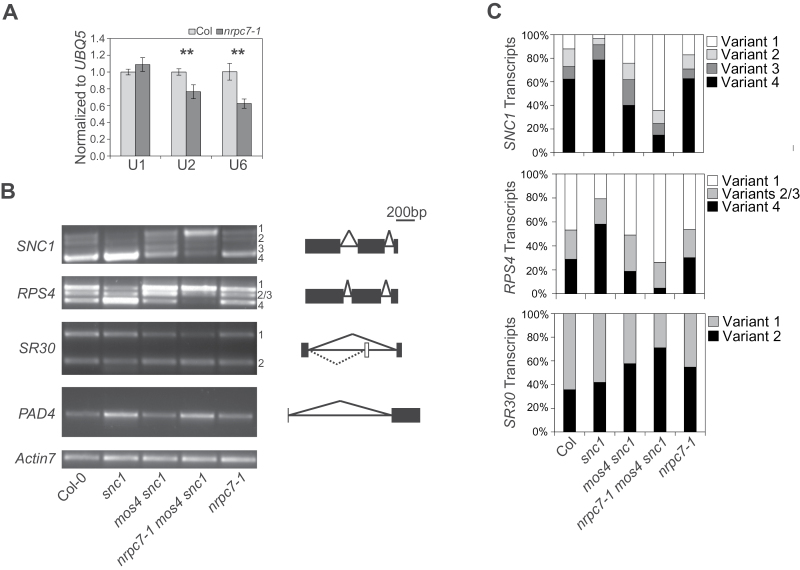
Splicing defects in *nrpc7-1*. (A) Quantitative real-time qPCR was used to determine the expression of Pol II-transcribed U1 and U2 snRNAs, as well as Pol III-transcribed U6, relative to UBQ5. Bars represent the averages of three technical replicates of two biological replicates ±SD. **P≤0.01. (B) An analysis of *SNC1*, *RPS4*, *SR30*, and *PAD4* splicing patterns in the indicated genotypes was performed using RT-PCR. Transcripts were amplified using 40 cycles. Numbers indicate transcript variants from largest to smallest. Schematic diagrams of the expected splicing events are shown to the right, with horizontal lines representing introns, black boxes representing exons, and white boxes representing alternatively retained exons that result in a premature stop codon. (C) Quantification of the alternative transcript variants in (B) across genotypes. Band intensities were quantified using ImageJ.

In yeast, Rpc25 is required for Pol III transcription initiation (Zaros and Thuriaux, 2005). To assess whether Pol III function is affected by the *nrpc7-1* mutation, we used real-time qPCR to determine whether expression of U6, a snRNA component of the spliceosome that is known to be transcribed by Pol III (Waibel and Filipowicz, 1990), is different in *nrpc7-1* than in wild type. Relative to the expression levels of the Pol II-transcribed ‘housekeeping’ gene *UBQ5*, U6 expression is significantly lower in *nrpc7-1* (Fig. 4A). To examine whether the *nrpc7-1* mutation has a general effect on spliceosomal snRNA biosynthesis, the accumulations of Pol II-transcribed U1 and U2 snRNAs were also examined. While U1 accumulation is wild type-like, U2 expression is significantly reduced in *nrpc7-1*. This is likely due to an indirect effect of altered Pol III function on Pol II-transcribed genes.

The reduced expression of the spliceosome components U6 and U2 leads us to hypothesize that pre-mRNA splicing might be affected by the *nrpc7-1* mutation. Specifically, as *nrpc7-1* was isolated in our screen for *snc1* enhancers, we hypothesized that the mutation may affect the excision of introns from the *SNC1* pre-mRNA transcript. The alternative splicing of a number of plant NLR-encoding genes, including *SNC1*, is known to affect their function in plant immunity (Yi and Richards, 2007; Xu *et al.*, 2011). For *SNC1*, the second and third introns may be either retained or removed; therefore we used primers spanning these two introns to amplify the *SNC1* transcript variants. A dramatic accumulation of the largest transcript variant (with both introns retained) was observed in *nrpc7-1 mos4 snc1*, although the *SNC1* splicing pattern in the *nrpc7-1* single mutant was indistinguishable from that observed in wild type (Fig. 4B and Supplementary Fig S3C). Alternative splicing defects were also observed in *nrpc7-1 mos4 snc1* for *RESISTANT TO PSEUDOMONAS SYRINGAE 4* (*RPS4*), another NLR-encoding gene, and to a lesser degree for *SR30*, which encodes a serine/arginine-rich RNA-binding protein and is known to be alternatively spliced (Fig. 4B). The relative proportions of the transcript variants in the genotypes examined are shown in Fig. 4C. These data reveal significant alternative splicing defects caused by the Pol III subunit mutation.

To determine whether this splicing defect occurs at the level of basal splicing, transcripts of a gene that is not alternatively spliced (*PHYTOALEXIN-DEFICIENT 4*; *PAD4*) were also examined. No difference from the wild type splicing pattern was detected (Fig. 4B). Both *snc1* and *nprc7-1 mos4 snc1* accumulated higher levels of *PAD4* compared with wild type, which is consistent with previous reports that *PAD4* is a defence-induced gene (Glazebrook 2001), whose expression is expected to be upregulated in autoimmune mutants.

NLR-mediated signalling is often regulated by modulating transcription and/or translation of NLRs. As such, we examined whether *SNC1* expression and protein accumulation are affected by the *nrpc7-1* mutation. *SNC1* expression was found to be slightly reduced in *nrpc7-1* as compared with wild type, while SNC1 protein levels were wild type-like (Supplementary Fig. S3). Similarly, the accumulation of SNC1 in *nrpc7-1 mos4 snc1* was not dramatically higher than that observed in *mos4 snc1*. Taken together, these data indicate that *SNC1* alternative splicing, but not overall gene expression or translation, is affected by the *nrpc7-1* mutation.

### The *nrpc7-1* single mutant does not have altered immune responses

Since alterations in the splicing of *SNC1* were observed in the *nrpc7-1 mos4 snc1* triple mutant but not the *nrpc7-1* single mutant, we predicted that *nrpc7-1* may not exhibit the enhanced disease resistance observed in the triple mutant (Fig. 1D). We challenged *nrpc7-1* with the oomycete pathogen *H*.*a*. Noco2 and the bacterial pathogen *Pseudomonas syringae* pv. *maculicola* ES4326, and found no statistically significant difference in response from that observed in wild type plants (Supplementary Fig. S4). These data support our hypothesis that the retention of introns in the *SNC1* transcript in the *nrpc7-1 mos4 snc1* confers enhanced disease resistance, and is likely the reason why this mutation was isolated from our *snc1* enhancer screen.

### *nrpc7-1* has global defects in RNA levels

**Fig. 5.**
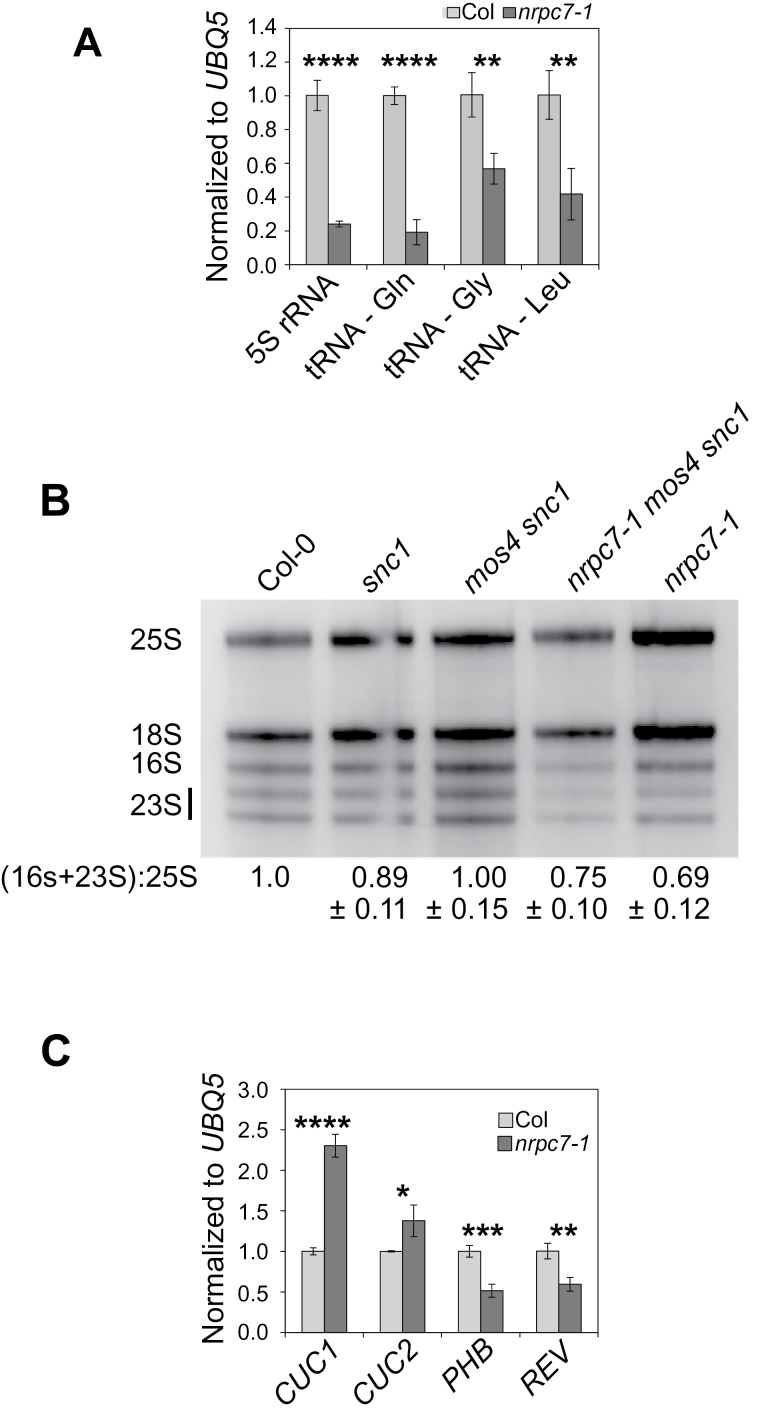
Global RNA defects in *nrpc7-1*. (A) Expression of several representative Pol III-transcribed RNAs was examined in Col-0 and *nrpc7-1* using qPCR. Bars represent the averages of three technical replicates of two biological repeats ±SD. (B) The proportions of rRNAs in the noted genotypes were compared by running total RNA extracted from seedlings grown on MS medium for 12 d on a 2% agarose gel. Band intensities were quantified using ImageJ, and the intensities of chloroplast rRNA (16S and 23S) relative to Pol I-transcribed rRNA (25S) were compared between genotypes for three biological replicates ±SD. (C) The accumulation of *CUC1*, *CUC2*, *PHB*, and *REV* transcripts was examined using quantitative real-time qPCR. Bars represent the averages of three technical replicates of two biological replicates ±SD. *P≤0.05; **P≤0.01; ***P≤0.001; ****P≤0.0001.

Pol III transcribes tRNA, 5S rRNA, and assorted other non-coding RNAs. To further explore how Pol III function is affected by the *nrpc7-1* mutation, we examined the expression of a variety of RNAs by qPCR (Fig. 5A). Relative to *UBQ5*, 5S rRNA and three representative tRNAs (coding for Gln, Gly, and Leu, respectively) showed significantly reduced accumulation in *nrpc7-1* compared with wild type. In addition, when total RNA was run on a 2% agarose gel, altered relative proportions of the various rRNAs were consistently observable in association with the *nrpc7-1* allele (Fig. 5B). Relative to Pol I-transcribed 25S rRNA, there appears to be a lower abundance of chloroplast 16S and 23S rRNA associated with the *nrpc7-1* allele. These data suggest that in addition to Pol III transcribed genes, the *nrpc7-1* mutation also affects abundance of other RNAs, likely through indirect mechanisms.

Small RNA libraries were then prepared from *BG2* plants (Col-0 with the *pPR2-GUS* reporter gene construct that is present in the *nrpc7-1* background) and two independently isolated *nrpc7-1* single mutant lines. Analysis of these small RNA libraries indicated that a number of miRNAs are differentially expressed in the mutant. Those miRNAs that exhibited a two-fold or greater change in expression are shown in Supplementary Fig. S5A. To validate these results, three representative miRNAs were selected for northern blot analysis. Although the data from the small RNA libraries indicated that expression of both *miR159* and *miR166* is reduced in *nrpc7-1* while *miR398* expression is increased, no significant alterations in the levels of these miRNAs were consistently observed via northern blotting (Supplementary Fig. S5B), suggesting that any differences that exist between the mutant and wild type are too subtle to be detected by this method.

The general disruption in RNA equilibrium combined with the striking serrated leaf phenotype and dwarf morphology of the *nrpc7-1* mutant led us to hypothesize that the expression of (i) the *CUP-SHAPED COTYLEDONS* (*CUC*) genes, which are targeted by *miR164* (Mallory *et al.*, 2004), and (ii) the HOMEODOMAIN-LEUCINE ZIPPER (HD-ZIP) genes, which are targeted by *miR165*/*166* (Rhoades *et al.*, 2002), might be altered in the mutant. Expression of a *CUC2* transcript resistant to *miR164* cleavage was previously shown to result in enhanced leaf serration; the same morphological phenotype was observed in plants containing loss-of-function mutations in the Pol II-transcribed *MIR164* (Nikovics *et al.*, 2006). Overexpression of either *miR165* or *miR166* results in reduced expression of the HD-ZIP genes, which corresponds with dwarf morphology and altered rosette leaf morphology that is similar to that observed in *nrpc7-1* (Jung and Park, 2007).

Real-time qPCR was used to determine that *CUC1* and *CUC2* accumulation is elevated in *nrpc7-1* (Fig. 5C), although no alteration in *miR164* levels were observed in *nrpc7-1* based on our small RNA library data. Expression of the HD-ZIP genes *PHABULOSA* (*PHB*) and *REVULOTA* (*REV*) was found to be decreased in *nrpc7-1* (Fig. 5C). No changes in *miR165* levels were observed in *nrpc7-1*, and although *miR166* expression was elevated in the mutant according to the small RNA library sequencing data (Supplementary Fig. S5A), no detectable change in *miR166* levels was consistently measurable by northern blotting (Supplementary Fig. S5B). The altered expressions of *CUC1*, *CUC2*, *PHB*, and *REV* in the absence of detectable changes in *miR164*, *miR165*, and *miR166* abundances suggests that in *nrpc7-1* the activity of a number of small RNAs may be affected; alternatively, the *nrpc7-1* mutation may indirectly affect Pol II-mediated transcription of certain genes, including *CUC1*, *CUC2*, *PHB*, and *REV*, although the mechanism behind this specificity is unclear. Many of the RNAs that seem to be differentially expressed in *nrpc7-1* are not transcribed by Pol III (Fig. 5), suggesting that this mutation results in a disruption of the global RNA equilibrium and homeostasis.

### NRPC7 localizes to the nucleus

**Fig. 6.**
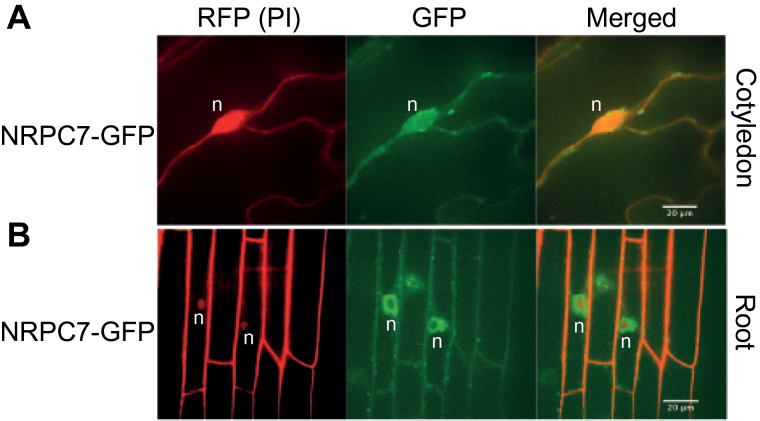
Subcellular localization of NRPC7-GFP. NRPC7-GFP as observed by confocal microscopy in cells from the cotyledon (A) and root (B) from Arabidopsis seedlings grown for 12 d on 1/2 MS medium. Propidium iodide (red) was used to stain the cell wall and nuclei. Scale bars represent 20 µm. n, nucleus.

As part of the Pol III complex, NRPC7 is predicted to localize to the nucleus. To examine its localization, we used the complementing *nrpc7-1* lines containing the transgene with *NRPC7* fused to *GFP* under the control of the native promoter, described above. We analyzed cotyledon and root tissue using confocal microscopy, and GFP fluorescence was visible throughout the nucleus as it co-localized with nuclei stained with propidium iodide (Fig. 6). Additionally, there appeared to be intense fluorescent foci within the nucleus and along the plasma membrane.

### *nrpc7-1* has pleiotropic developmental defects

**Fig. 7.**
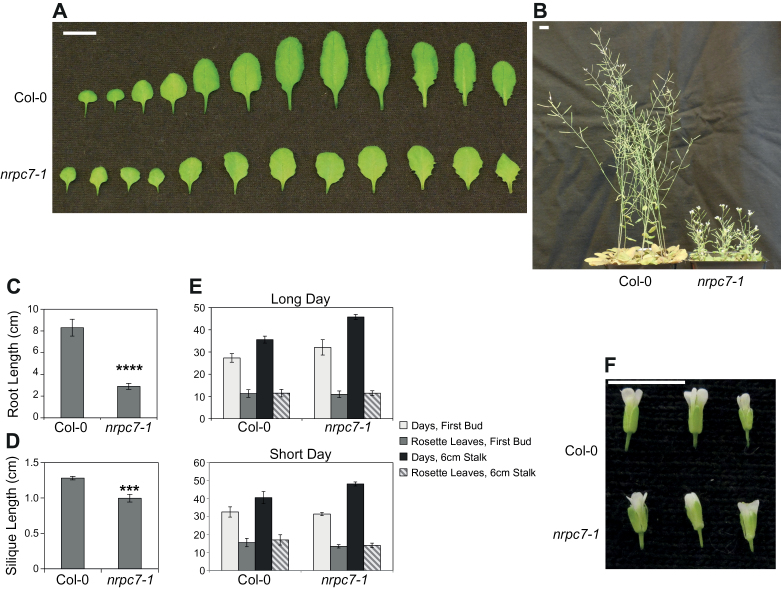
Developmental defects of the *nrpc7-1* mutant. (A) Morphology of rosette leaves from wild type and *nrpc7-1* plants at bolting. Bar indicates 1cm. (B) Morphology of soil-grown plants 8 weeks post-germination. Scale bar represents 1cm. (C) Comparison of root length in centimetres between wild type and *nrpc7-1*. Seedlings were grown vertically on ½ MS for 14 d. Bars represent five replicates ±SD. ****P≤0.0001. (D) Comparison of silique length between wild type and *nrpc7-1*. Siliques were harvested from mid-level of the primary stem for each plant. Bars represent three replicates of five siliques per plant ±SD. ***P≤0.001. (E) Four different approaches were used to investigate flowering time in wild type and *nrpc7-1* plants under both short day and long day conditions. (F) Morphology of wild type and *nrpc7-1* flowers. Scale bar indicates 0.5cm.

The roles various small RNAs play in the regulation of plant development have been well studied. As *nrpc7-1* has large impacts on small RNA levels and, potentially, RNA activities, we examined the developmental phenotypes of the mutant. As described earlier, *nrpc7-1* has serrated leaves (Figs 3A and 7A), and its growth is stunted (Fig. 7B). When grown on half-strength MS medium, *nrpc7-1* plants also have significantly shorter roots than wild type plants (Fig. 7C). The siliques of *nrpc7-1* were consistently found to be smaller (Fig. 7D). Flowering time was measured using several different assays, but a significant difference between *nrpc7-1* and wild type was only observed when measuring the number of days until the primary stalk reached 6cm (Fig. 7E), which is likely a reflection of the restricted growth of the mutant rather than an actual delay in flowering time. While the number and arrangement of the floral organs are wild type-like, the texture of the sepals is bumpy and irregular (Fig. 7F). These results show that the *nrpc7-1* mutation is associated with a number of pleiotropic developmental defects.

## Discussion

We demonstrated that Pol III function is altered by the partial loss-of-function mutation *nrpc7-1* by showing that the expressions of Pol III-transcribed U6 snRNA, 5S rRNA, and a number of tRNAs are reduced in the mutant (Figs 4A and 5A). Pol II-transcribed U2 snRNA, but not U1 snRNA, also had reduced expression in *nrpc7-1* (Fig. 4A), indicating that the transcriptional defects in the mutant extend to genes not directly transcribed by Pol III. The decreased accumulation of U6 and U2 snRNA led us to hypothesize that spliceosome functionality is impaired in *nrpc7-1* and that alternative splicing of *SNC1* is consequently affected, thereby explaining why this mutation was isolated from a screen for enhancers of the autoimmune mutant *snc1*. Indeed, *SNC1* splicing is defective in the *nrpc7-1 mos4 snc1* triple mutant background, in that there is a dramatic accretion of a transcript variant that retains both the second and third introns (Fig. 4B, C). A similar pattern was observed for the NLR-encoding gene *RPS4*. The second intron of *SNC1* contains an in-frame premature stop codon, and thus retention of this intron should yield a truncated version of the protein. Previous reports have shown that an accumulation of the N-termini of TNL proteins is sufficient to activate cell death and immunity (Weaver *et al.*, 2006; Swiderski *et al.*, 2009). This finding, combined with our data showing that transcription and translation of *SNC1* are not enhanced by the *nrpc7-1* mutation (Supplementary Fig. S3A, B), suggests that the modification in *SNC1* splicing could be the primary cause of the *snc1* enhancing effects of the *nrpc7-1* mutation in the *mos4 snc1* background (Fig. 1).

It is notable that the *nrpc7-1* single mutant does not differ from wild type in either alternative splicing (Fig. 4B, C) or disease resistance (Supplementary Fig. S4). This suggests that the *mos4* mutation is required in the genetic background for the *nrpc7-1*-associated splicing defects to become obvious. MOS4 is an integral component of the evolutionarily conserved MOS4-associated complex (MAC) that functions together with the spliceosome to regulate pre-mRNA splicing (Johnson *et al.*, 2011). Mutations in *mos4* and other MAC components have previously been shown to affect the alternative splicing of both *SNC1* and *RPS4* (Xu *et al.*, 2012), and are associated with a suppression of *SNC1*-dependent immune signalling (Palma *et al.*, 2007). In this study we demonstrated that there is an increased accumulation of the intron-retaining *SNC1* transcripts in *mos4 snc1* compared with *snc1* (Fig. 4C). However, this accumulation is radically enhanced in the *nrpc7-1* triple mutant. One possible explanation for these results is that the *mos4* mutation and, to a lesser extent, the *nrpc7-1* mutation individually disrupt splicing efficiency, reducing the pool of the functional full-length *SNC1* transcript variant with both introns excised but not increasing the production of alternative variants beyond the threshold required for immune activation. However, when these two mutations are combined in the *snc1* background, spliceosome activity is markedly disturbed and the accumulation of intron-retaining *SNC1* transcript variants is sufficiently high to yield enough truncated SNC1 to activate defence responses.

In addition to the splicing defects observed in *nrpc7-1*, the accumulations of rRNAs and tRNAs appear to be considerably distorted (Fig. 5A, B). Ribosomal protein gene dosage was recently found to have an effect on embryonic stem cell differentiation in mice (Fortier *et al.*, 2015), indicating that alterations in the abundance of ribosome components can dramatically alter developmental progression. Homozygous *nrpc7-1* plants exhibit certain phenotypes that may be associated with impaired stem cell differentiation including short roots (Fig. 7C) and delayed emergence of the first true leaves. This suggests that the sensitivity of ribosome function to changes in its subunit levels, as well as its role in regulating stem cell differentiation, may be conserved in plants, although the data in support of this are preliminary and additional experiments are required to fully explore this hypothesis.

We also detected a reduction in the accumulation of the chloroplast 16S and 23S rRNAs relative to Pol I-transcribed 25S rRNA (Fig. 5B). A similar rRNA abundance pattern was recently reported for *atybeY-1*, a mutant allele of an endoribonuclease required for chloroplast rRNA processing and development that also exhibits pale green leaves and delayed development (Liu *et al.*, 2015). Although the mechanism by which alterations in a Pol III subunit result in changes to the transcriptional regulation of Pol I-transcribed genes and the chloroplast genome is unclear, the light green colour of the *nrpc7-1* single mutant (Fig. 3A) further suggests that this mutation may be associated with impaired chloroplast function.

The serrated leaf phenotype observed in *nrpc7-1* is likely linked to its elevated expression of *CUC1* and *CUC2* (Fig. 5C), which could be a result of reduced *miR164* activity in the mutant background or an indirect effect of the *nrpc7-1* mutation on Pol II function. There may also be a link between the *nrpc7-1* mutant morphology and the decreased expression of the HD-ZIP genes (Fig. 5C). Other studies have demonstrated tentative links between the transcriptional activities of Pol II and Pol III. One study identified areas of the genome where protein-coding genes on one DNA strand overlapped with tRNA-coding genes on the opposite strand, and that their rates of transcription by Pol II and Pol III, respectively, were negatively correlated (Lukoszek *et al.*, 2013). Another study found that human *RPPH1* is transcribed by both Pol II and Pol III, and identified a number of transcriptional activators that associate with both Pols (Faresse *et al.*, 2012). Our data show that a disturbance in Pol III function affects the expression of non-Pol III-transcribed RNAs, indicating that the role of Arabidopsis Pol III in transcriptional regulation is more complex than previously assumed.

There are 12 core subunits of Arabidopsis Pol III, each of which has a homologue or is itself also a component in Pols I, II, IV, and V (Haag and Pikaard, 2011; Ream *et al.*, 2015). There are also subunits specific to individual Pols. *NRPC7* encodes a core Pol III subunit with homologues in each of the other Pols, and shares significant sequence similarity with Rpc25 proteins from other model organisms (Supplementary Fig. S1). However, Arabidopsis *NRPC7* failed to complement a temperature-sensitive *rpc25* yeast knockout line (Supplementary Fig. S2), suggesting that the functional conservation of this protein by itself between yeast and plants is limited. This is not entirely unprecedented. Rpc25 is known to form a dimer with Rpc17 within the Pol III complex (Siaut *et al.*, 2003). The protein–protein interaction surface of NRPC7 may be sufficiently evolutionarily divergent so as to prohibit it from dimerizing with yeast Rpc17. Although the function of the protein complex is conserved, an individual component of the complex may still be divergent enough that it fails to complement a knockout of its orthologue in a distant organism.

In summary, we have demonstrated that a perturbation in Pol III function results in modified gene splicing as well as alterations in the abundances and potentially activities of a number of RNA molecules. These effects extend to several RNAs reported to be transcribed by other polymerases, revealing a novel role for Pol III in modulating the expression of a larger complement of genes than previously described. Moving forward, the partial loss-of-function *nrpc7-1* mutant provides a unique tool for performing other functional analyses of Pol III.

## Supplementary data

Supplementary data are available at *JXB* online.

Figure S1. Sequence alignment of RPC25 from a broad range of species, based on BLAST analysis.

Figure S2. Yeast complementation with *NRPC7*.

Figure S3. *SNC1* gene and protein expression in *nrpc7*.

Figure S4. Immune characterization of *nrpc7-1* single mutant plants.

Figure S5. RNA defects in *nrpc7-1*.

Supplementary Data
