# Supplementary material for: A partial loss-of-function mutation in an Arabidopsis RNA polymerase III subunit leads to pleiotropic defects
Source: J Exp Bot. 2016 Feb 10;67(8):2219–30. doi: 10.1093/jxb/erw020 (PMC4809280; doi:10.1093/jxb/erw020)
Supplement: Supplementary Data [file supp_67_8_2219__index.html]

A partial loss-of-function mutation in an Arabidopsis RNA polymerase III subunit leads to pleiotropic defects — Supplementary Data 

# A partial loss-of-function mutation in an Arabidopsis RNA polymerase III subunit leads to pleiotropic defects

## Supplementary Data

Data files

- Supplementary\_Figure\_1.eps - Supplementary Data
- Supplementary\_Figure\_2.eps - Supplementary Data
- Supplementary\_Figure\_3.eps - Supplementary Data
- Supplementary\_Figure\_4.eps - Supplementary Data
- Supplementary\_Figure\_5.eps - Supplementary Data
